# ALG-2 participates in recovery of cells after plasma membrane damage by electroporation and digitonin treatment

**DOI:** 10.1371/journal.pone.0204520

**Published:** 2018-09-21

**Authors:** Jonas M. la Cour, Pernille Winding Gojkovic, Sophie E. B. Ambjørner, Jonas Bagge, Simone M. Jensen, Svetlana Panina, Martin W. Berchtold

**Affiliations:** Department of Biology, University of Copenhagen, Copenhagen, Denmark; Augusta University Medical College of Georgia, UNITED STATES

## Abstract

The calcium binding protein ALG-2 is upregulated in several types of cancerous tissues and cancer cell death may be a consequence of ALG-2 downregulation. Novel research suggests that ALG-2 is involved in membrane repair mechanisms, in line with several published studies linking ALG-2 to processes of membrane remodeling and transport, which may contribute to the fitness of cells or protect them from damage. To investigate the involvement of ALG-2 in cell recovery after membrane damage we disrupted the *PDCD6* gene encoding the ALG-2 protein in DT-40 cells and exposed them to electroporation. ALG-2 knock-out cells were more sensitive to electroporation as compared to wild type cells. This phenotype could be reversed by reestablishing ALG-2 expression confirming that ALG-2 plays an important role in cell recovery after plasma membrane damage. We found that overexpression of wild type ALG-2 but not a mutated form unable to bind Ca^2+^ partially protected HeLa cells from digitonin-induced cell death. Further, we were able to inhibit the cell protective function of ALG-2 after digitonin treatment by adding a peptide with the ALG-2 binding sequence of ALIX, which has been proposed to serve as the ALG-2 downstream target in a number of processes including cell membrane repair. Our results suggest that ALG-2 may serve as a novel therapeutic target in combination with membrane damaging interventions.

## Introduction

The EF-hand Ca^2+^-binding protein ALG-2 has been implicated in a variety of cellular processes including apoptosis, proliferation and protein trafficking among others (reviewed in [1,2]). Originally, ALG-2 was considered a proapoptotic protein based on its discovery as a mediator of T-cell apoptosis [3]. Further early findings indicated that ALG-2 may play a proapoptotic role in ER stress induced cell death of human embryonic kidney cells and mouse embryonic fibroblasts [4], in programmed cell death of cervical motoneurons in chick embryos [5] as well as in uveal melanocytes possibly preventing the development of melanoma [6]. Yet, a mouse ALG-2 knock-out model did not support a role for ALG-2 in apoptosis [7] and it is well documented that ALG-2 may play important roles in promoting proliferation as it was found overexpressed in certain tumors and its downregulation led to inhibition of cell proliferation and caspase-dependent cell death [8–10].

Whereas no direct mechanistic role for ALG-2 in cancer cell viability has been identified, recent discoveries have linked ALG-2 to membrane vesicle traffic and cargo packaging via its interaction with Sec31A [11,12]. A number of other well described ALG-2 targets are physically and/or functionally associated with the plasma or organelle membranes (reviewed in [2]) indicating a role of ALG-2 in membrane linked processes. ALIX, also called AIP1 was the first ALG-2 binding protein identified [13,14]. It has been found to be associated with components of ESCRT important for a plethora of cellular processes associated with membrane remodeling, including endosome formation, fusion of autophagosomes and amphisomes with lysosomes as well as retrovirus budding among others (reviewed in [15]).

This study aimed to shed further light on the proposed cell protective function of ALG-2 with regard to its effect on cell viability following membrane damage [16]. We tested whether ALG-2 expression may be beneficial for recovery of cells after electroporation- and digitonin-induced plasma membrane damage using a novel ALG-2 knock-out system in a chicken B cell line and ectopic overexpression of ALG-2 in human cancer cells and whether the function of ALG-2 in this process is Ca^2+^-dependent and involves ALIX interaction.

## Materials and methods

### Reagents

Polyclonal antibodies against ALG-2 were raised in rabbits against full length recombinant ALG-2 as described in [17]. ERK-1 antibodies were from Santa Cruz (K-23) and horseradish peroxidase coupled secondary anti-rabbit-antibody from DAKO, Denmark.

The peptides used were >95% pure and either with or without N-terminal TAMRA label.

ALIX peptide: QGP**PY**PTYPGYPGYCQ, ALIX mutant peptide: QGP**AA**PTYPGYPGYCQ; control peptide (unrelated) Syntide 2: PLARTLSVAGLPKK. Mutated residues are shown in red.

The ALIX peptides (wt and mutant, which has been shown not to be able to interact with ALG-2) [18] as well as the TAMRA labeled ALIX peptides were purchased from Proteogenix (Schiltigheim, France) and the Syntide 2 peptide was from LKT laboratories Inc. (St. Paul, MO, USA).

Blasticidin S, puromycin, zeocin, digitonin and trypan blue were purchased from Sigma and the ECL reagent was purchased from GE Healthcare Amersham.

The EGFP expression plasmids are described in [19] with the exception of the ALG-2 isoform producing a protein that lacks Gly^121^/Phe^122^, EGFP-ALG-2^ΔGF^ [20] which was generated by conventional PCR based mutagenesis.

### Cell culturing

Chicken B cell line DT-40 cells (kind gift from Prof. T. Kurosaki, Osaka) were routinely cultured in RPMI 1640 medium supplemented with 10% fetal bovine serum, 1% chicken serum, 2 mM L-glutamine and penicillin/streptomycin, at 40°C in humidified 5% CO_2_ atmosphere. HeLa cells, purchased from ATCC, were grown in DMEM medium with 10% fetal bovine serum, 2 mM L-glutamine, penicillin/streptomycin, at 37°C in humidified 5% CO_2_ atmosphere.

### Generation of ALG-2 deficient DT-40 cells

A λFIXII chicken spleen genomic library (Stratagene) kindly provided by Prof. T. Kurosaki (Osaka University) was screened with a cDNA probe of chicken *PDCD6*. A clone containing 5 out of 6 exons (exons 2–6) of the *PDCD6* gene was selected for further generation of the targeting vector. A 5’ flanking region of 0.9 kb, containing intron 1 sequences and a 3’ flanking region of 2.5 kb downstream of exon 4 were subcloned into pBluescript II SK (-). The bsr, puro and zeo drug resistance cassettes were inserted in the above vector resulting in the constructs pAlg2-Bsr, pAlg2-Puro and pAlg2-Zeo. Upon integration of any of these constructs into the chicken genome exons 2, 3 and 4 of the *PDCD6* gene (encoding amino acid residues 21–109 of chicken ALG-2) were deleted by homologous recombination. The *PDCD6* gene disruption was done sequentially by first transfecting parental DT-40 cells with 10 μg of the linearized pAlg2-Bsr by electroporation (550V, 25μF). Drug resistant clones were selected in the presence of 50 μg/ml blasticidin S HCl. The clones were analyzed by Southern blotting. A *PDCD6*^-/+/+^ clone was further transfected with the pAlg2-Puro construct. *PDCD6*^-/-/+^ clones were selected with 0.5μg/ml puromycin in the presence of blasticidin. Finally, the cells were transfected with the pAlg2-Zeo construct and selection was done with 1 mg/ml zeocin in the presence of blasticidin and puromycin.

### Southern blotting

Genomic DNA was purified from drug resistant clones using standard techniques. 10μg DNA were digested with *Nco*1 and run on a 1% TAE agarose gel, blotted onto a nylon membrane and probed with a ^32^P-labeled 0.9 kb PCR-generated *PDCD6* genomic probe containing intron 1 with no sequence homologies to other chicken genomic DNA.

### Ectopic expression of ALG-2 in the ALG-2 knock-out cells

The *PDCD6* cDNA encoding chicken ALG-2 was cloned into the *Kpn*1-*Xba*1 sites of the expression vector pcDNA3 containing the *neo* gene allowing for selection of geneticin resistant clones. DNA was linearized with *Nru*1 and used for transfection of the ALG-2 knock-out DT-40 cell line 17-2-11 by electroporation (124 V and 750 μF giving a field strength of 0.3 kV/cm).

### Transient transfection of HeLa cells

One day before the experiments, HeLa cells were transfected with the following constructs: EGFP (control), EGFP-ALG-2, EGFP-ALG-2^ΔGF^, EGFP-ALG-2^EF-1,-3^) in 96 well plates, 15.000 cells/well, using polyethylenimine (PEI, linear, MW 25.000) as a DNA carrier for transfection as described in [21,22]. The PEI/DNA ratio was 15:1.

### Induction of plasma membrane damage

Electroporation was used to induce a mechanical membrane damage in DT-40 cells [22]. Cells were grown to a density of 0.8-1x10^6^/ml, collected by centrifugation and washed in HBSS. 2x10^6^ cells resuspended in 100 μl HBSS were electroporated in a 0.1 cm cuvette with a pulse of 124V or 200V at 100 μF. The cells were immediately transferred to a 6-well plate containing pre-heated growth medium and propagated until further analysis. Control samples were handled identically without undergoing electroporation.

Digitonin was used to induce plasma membrane damage of HeLa cells as earlier described by Jimenez [23]. The cells were washed with PBS and 20 μl of full growth medium containing 50 μM digitonin was added per well of a 96 well plate in the presence or absence of 10 μM of ALG-2-binding peptide or control peptide. The cells were incubated at room temperature for 3 minutes. After removing digitonin, cells were washed with 100 μl of fresh medium per well and returned into a CO_2_ incubator until further analysis.

### HeLa cell viability measurements

To examine HeLa cell viability after plasma membrane damage by digitonin the number of EGFP expressing HeLa cells were recorded using an OPERA high content screening confocal microscope (Perkin Elmer) with a 20x NA 1.0 water immersion objective at different time points pre- and post digitonin treatment. EGFP fluorescence was excited at 488 nm and emission light was passed through a 565sp dichroic filter, followed by a 540/75 filter and captured using camera 1. TAMRA fluorescence was recorded using 568 nm excitation and emission was collected using a 600/40 filter, in camera 2. Images of ten regions of interest were collected per well. The number of fluorescent cells in each picture was counted manually and the cell viability was calculated as the ratio of fluorescent cells post-treatment by the number of fluorescent cells pre-treatment.

### Viability assessment using manual cell counting

Trypan Blue Solution was added to a DT-40 cell suspension at 0.2% (v/v) and cells were counted using a Bürker-Türk hemocytometer. Blue cells were considered dead and unstained cells–alive.

### Western blotting

Preparation of cell lysates and Western blotting were performed as described in [8,20]. After staining with anti-ALG-2 antibody, the membranes were stripped in a buffer containing 37.5 mM Tris-HCl, pH 6.8, 2% SDS and 1% beta-mercaptoethanol under rotation for 30 min at 55 ^o^C. The stripped membranes were re-probed with anti-ERK-1 antibody as a loading control.

### Statistical analysis

Statistical treatment of the data was performed using the GraphPad Prism 6.0 software.

## Results

### ALG-2 knock-out in DT-40 cells and ectopic ALG-2 expression

To investigate ALG-2 functions including its role in supporting maintenance of the plasma membrane integrity following damage the *PDCD6* gene was disrupted in DT-40 cells. The DT-40 B cell line was chosen for the complete genetic disruption of the *PDCD6* gene as these cells have a high homologous recombination rate making this approach feasible (reviewed in [22]).

**Fig 1:**
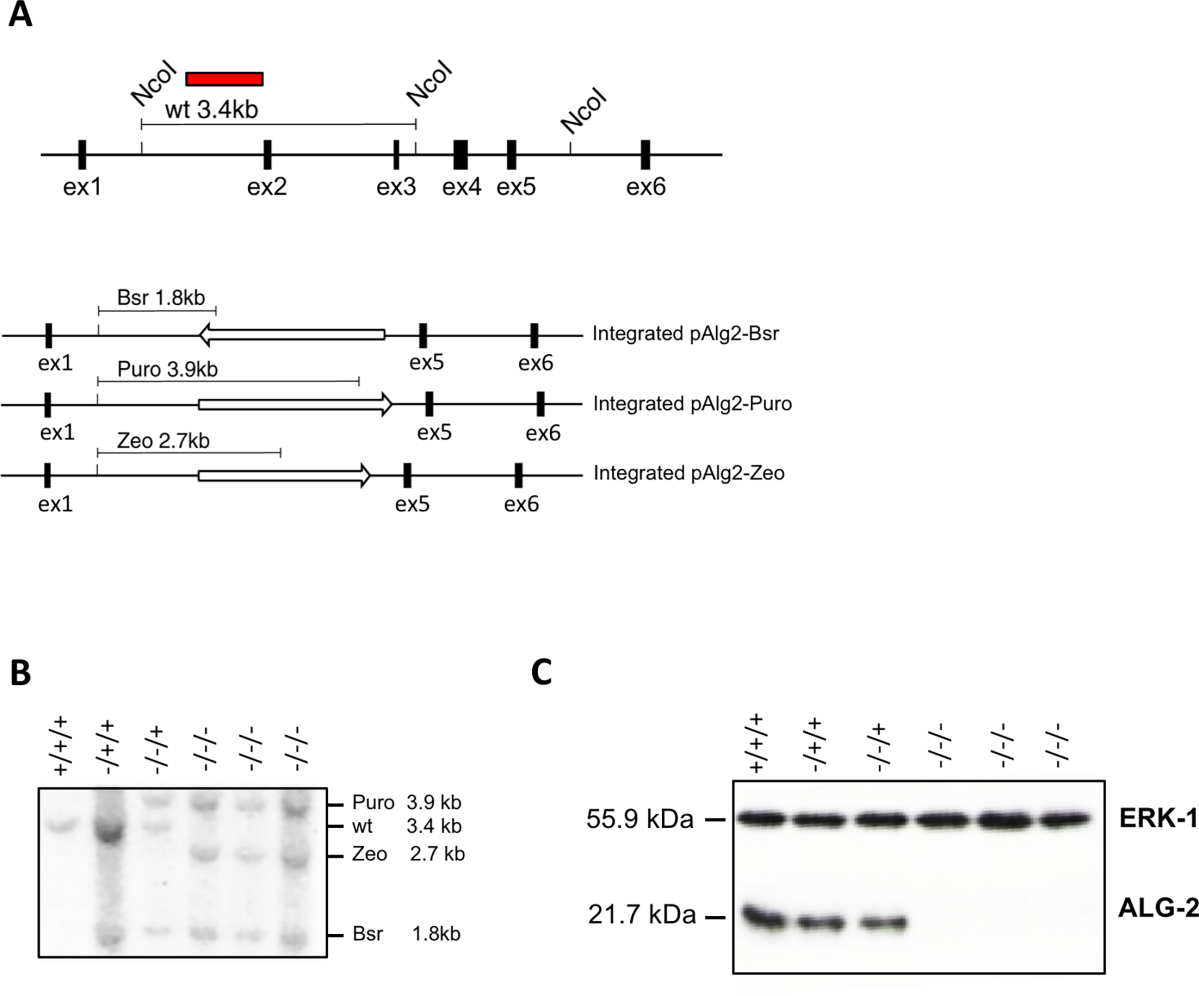
*PDCD6* gene knock-out in DT-40 cells and ectopic ALG-2 expression. **A.** *Top*: Strategy of *PDCD6* gene knock-out. The genomic organization of the chicken *PDCD6* gene; *Nco*1 restriction digestion of genomic DNA was used for Southern blot analysis, the sites within the *PDCD6* gene are indicated; the probe used for hybridization is shown as a red box; the size of the *Nco*1 digested DNA fragment to which it hybridizes in the wt cells is indicated above the gene structure. *Bottom*: the genomic situation after homologous recombination and the length of the expected *Nco*1 fragments. The open arrows indicate the drug resistance genes and their transcriptional orientation. **B.** Southern blot of genomic DNA from all the cell lines used and generated during *PDCD6* gene disruption hybridized with a chicken *PDCD6* cDNA probe. **C.** Western blot of protein extracts from the same cell lines as in **B** using a specific ALG-2 antibody and ERK-1 as a loading control.

The *PDCD6* gene is localized on chromosome 2 which is present in three copies in DT-40 cells [24]. We chose to eliminate exons 2, 3 and 4 of the *PDCD6* gene based on the fact that these exons encode the functional Ca^2+^ binding EF-hands 1 and 3. Three different drug resistance gene expression cassettes (blasticidin S, puromycin and zeocin) were used to replace sequences of *PDCD6* by homologous recombination (Fig 1A). The proper integration of these constructs was assessed by Southern blotting of DNA from the clones after digestion with *Nco*I followed by hybridization with a 0.9kb probe corresponding to the 5’ flanking region. Three clones having all three ALG-2 alleles disrupted were identified (Fig 1B). These clones did not express any detectable ALG-2 protein as shown by Western blot analysis (Fig 1C).

### Depletion of ALG-2 causes decreased viability following electroporation

**Fig 2:**
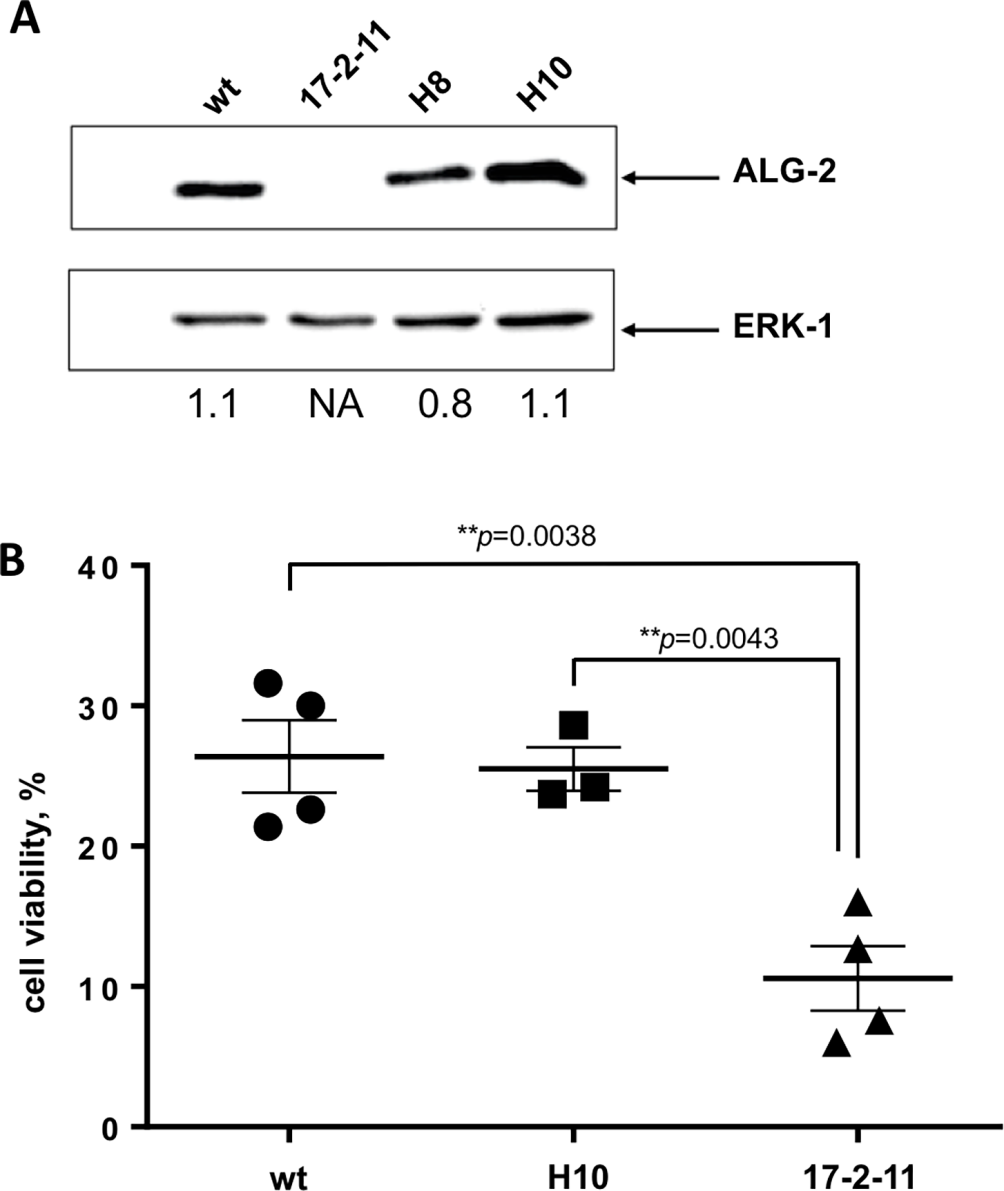
ALG-2 knock-out leads to reduced cell viability after electroporation induced membrane damage. **A.** Western blot analysis of cell extracts from DT-40 wt, an ALG-2 KO cell line 17-2-11, and two 17-2-11 clones, H8 and H10 transfected with an ALG-2 expressing vector. ERK-1 was used as a loading control. Numbers below represent the relative ALG-2 signal intensities normalized to the ERK1 signals. Quantification was performed using ImageJ software. **B.** DT-40 wt cells, an ALG-2 KO cell line 17-2-11 and a rescue clone H10 were exposed to electroporation in order to investigate the involvement of ALG-2 in cell survival after mechanical membrane damage. Manual cell counting was performed 24 hours post electroporation. Individual data from four independent experiments are shown. Means +/- SEM are indicated. The *p*-values (unpaired *t*-test) are indicated.

The selected ALG-2 knock-out (KO) cells were viable and their survival rate did not differ from wild type (wt) cells indicating that ALG-2 is not essential for DT-40 cell survival under normal growth conditions. Based on the proposed function of ALG-2 as a cell viability supporting protein as well as on the report on the involvement of ALG-2 in membrane repair after laser induced membrane damage [16] we thus investigated whether there would be a difference in cell viability between ALG-2 KO and wt cells when the cells experience a physical plasma membrane damage. To do this we used electroporation, an established method to induce plasma membrane lesions [25] yet not applied previously to investigate the role of ALG-2 in recovery after membrane damage. DT-40 cells were electroporated and cell viability was used as a marker for the cells capacity to recover after membrane damage (Fig 2). Measurement of cell viability 24 hours after electroporation in four independent experiments revealed that wt cells recovered significantly more effectively from electroporation than ALG-2 KO 17-2-11cells with an average wt cell viability of 26.4% versus 10.5% in KO cells. To analyze whether the phenotype detected in KO cells could be reversed we reestablished ALG-2 expression in the ALG-2 17-2-11 KO cell line by stably transfecting cells with a chicken ALG-2 expressing plasmid. The clone H10 with expression level in the range of DT-40 wt cells was selected for further analysis. The H10 cells were nearly as viable as the wt cells with an average viability of 25.5% 24 hours after electroporation indicating that reintroducing ALG-2 expression in the ALG-2 KO cell line 17-2-11 reversed the observed phenotype.

### Overexpression of ALG-2 increases the survival rate after digitonin -induced cell membrane damage

**Fig 3:**
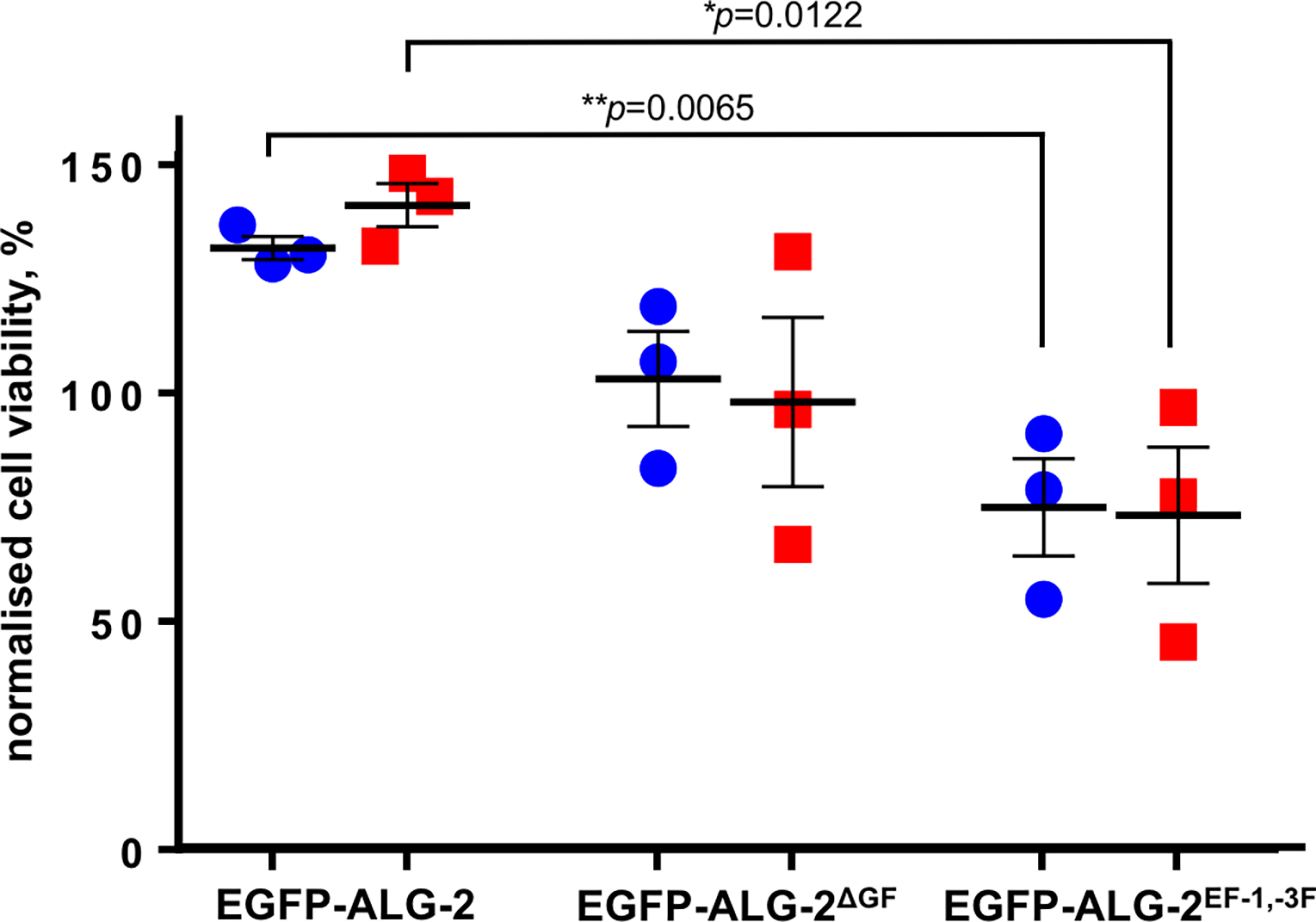
ALG-2 overexpression leads to increased cell viability after digitonin treatment. HeLa cells were transfected with four different constructs: EGFP (control), EGFP-ALG-2 (wt ALG-2), EGFP- ALG-2^ΔGF^ (ALG-2 short isoform missing Gly121 and Phe122), EGFP-ALG-2^EF-1, -3^ (ALG-2 protein with mutated high affinity Ca^2+^-binding sites). 50 μM digitonin was used to induce membrane damage. Cell viability was assessed by imaging and quantifying EGFP expressing cells one hour (blue symbols) and three hours (red symbols) post-treatment. Data were normalized to the EGFP transfected control set as 100%. Individual data from three independent experiments are shown. Means +/- SEM are indicated. The *p*-values (unpaired *t*-test) are indicated.

To further investigate the influence of ALG-2 on cell survival after plasma membrane damage of an adherent mammalian cell line of human origin, we treated HeLa cells transfected with ALG-2 expression constructs with digitonin and followed cell survival up to three hours after treatment (Fig 3). Overexpression of ALG-2 markedly increased cell viability after digitonin treatment compared to the EGFP control (~30% increase after 1 hour and 40% after 3 hours) whereas ectopic expression of the ALG-2 isoform lacking amino acids Gly^121^ and Phe^122^ and which is not able to bind ALIX [20], showed no effect. Interestingly, ALG-2 incapable of Ca^2+^ binding due to point mutations within EF-hand 1 and EF-hand 3, decreased cell viability compared to the EGFP control (~25% decrease after 1 hour and 27% after 3 hours). These findings indicate that ALG-2 may protect cells from death caused by plasma membrane damage and that the ALG-2 protective function is dependent on the propensity of ALG-2 to bind Ca^2+^. Ectopically expressed ALG-2, which is not able to bind Ca^2+^, may act as a dominant negative mutant by heterodimerizing with endogenous ALG-2 in our experiment.

### ALIX peptides interfere with cell recovery after membrane damage

**Fig 4:**
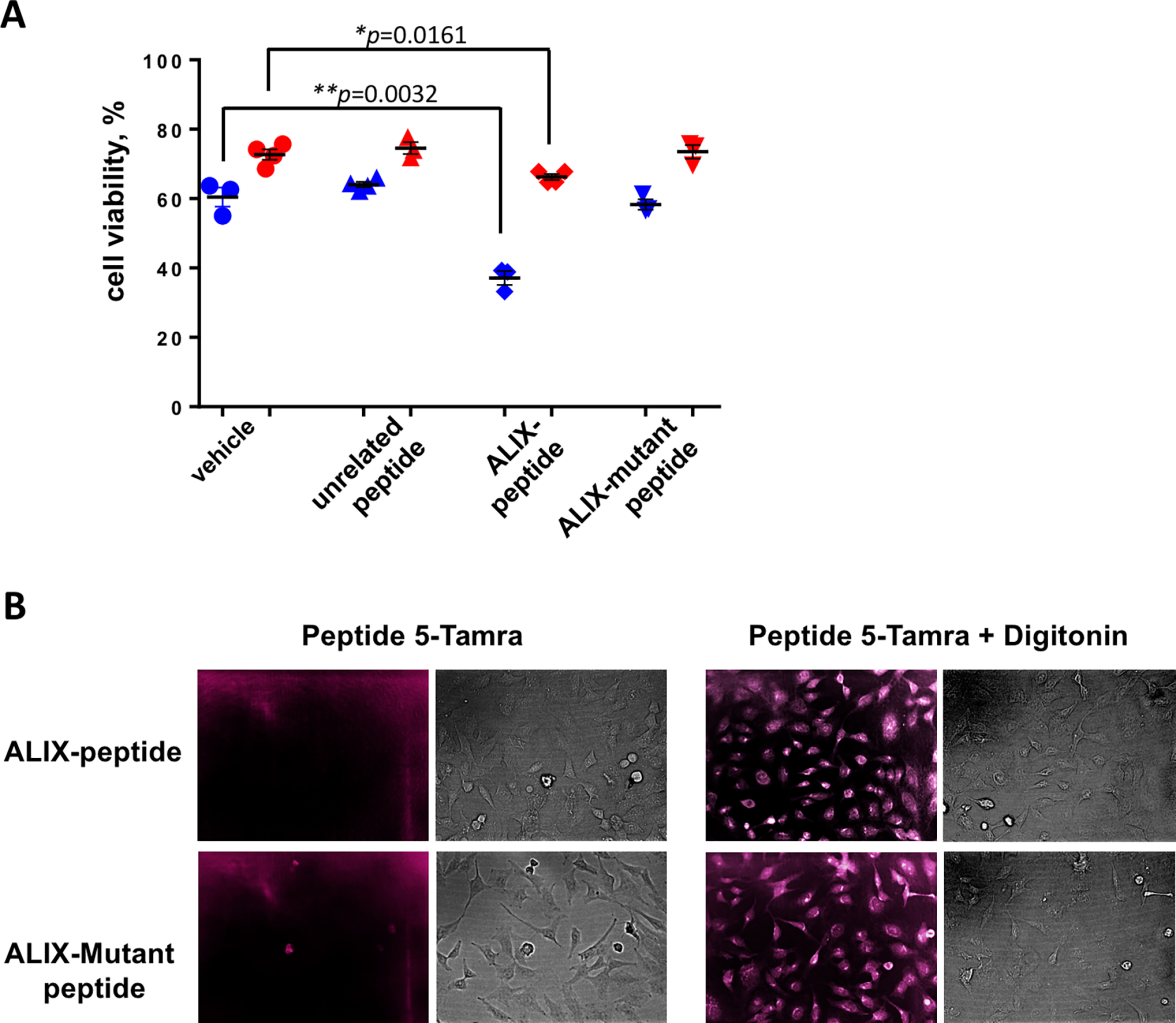
A peptide with the ALG-2 binding sequence of ALIX impairs the ALG-2 pro-survival function. **A.** HeLa cells expressing EGFP or EGFP-ALG-2 were treated with 50 μM digitonin in the presence of 10 μM ALIX peptide with ALG-2 binding sequence, ALIX mutant peptide incapable of ALG-2 binding, unrelated peptide or vehicle, as indicated. Cell viability was assessed by quantifying EGFP expressing cells (blue symbols) or cells expressing EGFP-ALG-2 (red symbols) one hour post-treatment. Individual data from at least three independent experiments are shown. Means +/- SEM are indicated. Statistical analysis was performed using unpaired *t*-test with Welch’s correction. **B.** Cellular uptake of TMRA-labeled ALIX peptides (*top panels*: wt ALG-2 binding ALIX peptide, *bottom panels*: mutated version incapable of ALG-2 binding) with the corresponding brightfield images in the absence (*left panel*) or the presence (*right panel*) of digitonin.

To investigate whether it is possible to suppress the protective activity of ALG-2 by blocking its interaction with ALIX, proposed to play a role in membrane repair [16], we treated HeLa cells transfected with EGFP or EGFP-ALG-2 with digitonin in the presence of a 10 μM synthetic peptide containing the ALG-2 binding sequence of ALIX, a mutated form not able to bind ALG-2 [18] and an unrelated peptide. The ALIX peptide significantly enhanced membrane damage-induced cell death as measured 1 hour after digitonin treatment of EGFP expressing cells (from ~60% to 37% viability), whereas the corresponding ALIX sequence mutated in two positions known to be crucial for ALG-2 binding did not affect cell viability (Fig 4A). This points to the importance of the ALIX-type binding site of ALG-2 [26] in the here described function of ALG-2, possibly involving the ESCRT protein complex as proposed by Scheffer et al. for laser induced membrane damage [16]. In contrast, cells transfected with EGFP-ALG-2 were affected by the ALG-2 binding peptide to a lesser degree pointing to the possibility that overexpression of ALG-2 may lead to sequestering of the peptide and counteract its inhibitory function. These data further indicate that ALG-2 plays a cell protective role following plasma membrane damage.

To test whether the synthetic peptides were internalized after digitonin treatment fluorescently labeled ALIX peptides (wt and mutated versions) were monitored by microscopy. Treatment of cells with digitonin led to intracellular presence of the labeled peptides whereas the peptides were not able to enter non-treated cells (Fig 4B).

## Discussion and conclusions

Here, we present data providing evidence for the function of ALG-2 in cell survival after cell membrane damage induced by electroporation and digitonin treatment based on the following main findings: (1) an engineered chicken B cell line lacking the *PDCD6* gene showed higher sensitivity to membrane damage by electroporation compared to the wild type cells and this phenotype could be reversed by reestablishing ALG-2 expression; (2) overexpression of ALG-2 in human cancer cells led to increased recovery after membrane damage caused by digitonin treatment.

Our results are in line with findings indicating that, despite ALG-2 discovery as a proapototic protein and data supporting this view (29–35), it may play important roles in cell survival mechanisms. In support of a cell viability maintaining function of ALG-2 a number of studies showed overexpression of ALG-2 in certain tumors and a cell protective function of ALG-2 [17] [8]. In a study of over 7000 tumor samples of various origin we found a significant upregulation of ALG-2 in mesenchymal tumors and downregulation of ALG-2 by siRNA in HeLa cells led to loss of cell viability [8], cell cycle arrest in the G2 phase and increased amount of early apoptotic and dead cells [10]. Sun et al. [27] showed that ALG-2 is highly expressed in patients with metastatic ovarian cancer and its expression level was shown to be positively correlated with disease progression suggesting that ALG-2 could be an independent predictor of progression free survival in this group of patients. In addition, Ariel et al. [28] identified ALG-2 as a biomarker candidate for invasion and progression of pulmonary adenocarcinoma as high ALG-2 expression correlated with poor survival prognosis. Recent work by Qin and collaborators [9] demonstrated that expression of ALG-2 is upregulated in breast cancer tissues and its expression is correlated with clinicopathological characteristics indicative of tumor malignancy. In addition, they showed that ALG-2 is positively involved in breast cancer growth and metastasis in mice and promotes cell proliferation, survival and motility *in vitro*. Their work indicates that upregulated ALG-2 may function by interfering with microtubule dynamics leading to chromosomal missegregation.

ALG-2 is an adaptor protein with the propensity to interact with a number of different targets and by this exerting an effect on important physiological processes including ER to Golgi vesicle transport and endosomal trafficking (reviewed in [1,2]). ALG-2 has been shown to interact with a variety of targets in different ways based on distinct binding pockets in ALG-2 recognizing different target sequences [29]. In mammalian cells, ALG-2 is present in two splice forms and occurs as dimers. The more abundant form contains two extra residues G121, F122 and has a different spectrum of interaction partners [20,26]. Interestingly, only this isoform is able to interact with ALIX. In addition, it is known that ALG-2 can form homo and heterodimers, which consist of the two splice forms [20]. It can be predicted that the functional outcome of the action of ALG-2 will depend on the composition of these dimers in the cell. Henzel [30] found that the homodimers of the long and the short isoform and the heterodimers displayed different dissociation constants which were also dependent on target interaction pointing to a further level of complexity. Interestingly, we found that when we overexpressed the short splice form the cell viability after digitonin-induced membrane damage was not affected (Fig 3) indicating that only the long splice form supports cell survival in our experiment. Further, ALG-2 forms dimers in the absence of Ca^2+^ with peflin, another penta EF hand protein, which may regulate the abundance of freely available ALG-2 in the cell [31] and /or cooperate with ALG-2 in controlling protein modification as shown for the ubiquitinylation of Sec31 by CUL3^KLHL12^ [12].

Even though we could show that ALG-2 interaction with the ALG-2 binding peptide of ALIX interferes with its pro-survival function it could well be that other ALG-2 targets known to play a role in membrane dynamics are important for cell recovery after plasma membrane damage. Examples are proteins associated with the ER and lysosome membranes, which both may be in indirect or direct contact with the plasma membrane under certain circumstances. One example is Sec31A, an ALG-2 binding partner, which is part of the COPII vesicle coat transporting cargos from the ER. ALG-2 has been shown to be functionally involved in regulating early events in ER to Golgi transport such as COPII budding and ERGIC formation [11,12,32]. In addition, ALG-2 directly binds to the lysosomal Ca^2+^ channel mucolipin-1 (MCOLN1, TRPML1) in a Ca^2+^ dependent way. Mutation of the ALG-2 binding sites in mucollipin-1 leads to a reduction of the propensity of MCOLN1 to participate in endosome membrane fusion indicating a regulatory role of ALG-2 in this process [33]. Furthermore, Li et al [34] showed that ALG-2 is the Ca^2+^ sensor for the MCOLN1 dependent centropetal movement of lysosomes as ALG-2 knock-out by CRISPR blocked the retrograde movement of lysosomes after treatment with the MCOLN1 activator ML-SA1.

Our data show that the effect of ALG-2 on the cell viability after membrane damage is Ca^2+^ dependent as a mutated version of this protein lacking the ability to bind Ca^2+^ did not support cell survival. In contrast, the mutant protein increased cell death indicating that it may form dimers with the endogenous ALG-2 suppressing the pro-survival function of the latter. In conclusion, our results support the hypothesis that ALG-2 is a prosurvival factor and plays an important role in cell recovery after membrane damage. However, our study does not provide evidence that the observed Ca^2+^ dependent activity of ALG-2 in cell recovery after membrane damage is the consequence of its involvement in the ESCRT mediated membrane shedding after damage (3). ALG-2 is known to have multiple targets playing a role in cellular processes not directly linked to membrane repair including e.g. regulation of Ca^2+^ signal transduction, protein trafficking, cytoskeletal organization and lysosomal functions, which all may support cell viability (reviewed in 4,5). In addition, it could be speculated that ALG-2 supports mechanisms to improve Ca^2+^ sequestering after the massive influx of this ion during membrane damage. These functions of ALG-2 may by themselves or in concert with others as well explain our observations.

Improving the efficiency of cell recovery after membrane damage by upregulating ALG-2 could provide a selective advantage for migrating cancer cells as they are continuously under mechanical stress. This has recently been shown to be the case for S100A11, another Ca^2+^ binding protein proposed to be involved in plasma membrane repair [35]. Further studies are needed to understand at the molecular level how ALG-2 is supporting cell recovery after membrane damage.
